# EXPANDING EDUCATIONAL OPPORTUNITIES IN GERONTOLOGY IN JAPAN

**DOI:** 10.1093/geroni/igad104.1092

**Published:** 2023-12-21

**Authors:** Noriko Tsukada, Nobuhiro Maeda, Katsuya Iijima

**Affiliations:** Nihon University, Setagata-city, Tokyo, Japan; NLI Research Institute, Chiyoda-ku, Tokyo, Japan; The University of Tokyo, Tokyo, Tokyo, Japan

## Abstract

There are two universities that offer Gerontology curriculum and confer Gerontology degrees in Japan, including J.F. Oberlin University and The University of Tokyo. J.F. Oberlin University started its Master’s program in 2002 and its doctoral program in 2004. The University of Tokyo endowed a Gerontology Research Division in 2006 and started its cross-departmental program in Gerontology in 2008. The endowed Gerontology Research Division was elevated to the Institute of Gerontology (IOG) in 2009. From April 2014 through March 2021, the Gerontology program, known as the Global Leadership Initiative for Age-Friendly Society (GLAFS) at The University of Tokyo, was funded by the Ministry of Education, Culture, Sports, Science and Technology. Today, The University of Tokyo now funds the program which was renamed World-leading Innovative Graduate Study Program in Gerontology: Global Leadership Initiative for Age-Friendly Society (WINGS-GLAFS). This paper first presents a brief history of Gerontology-related events in Japan, including foundational education proposals of The Science Council of Japan, then introduces Japan’s leading Gerontology education programs offered by J.F. Oberlin University and The University of Tokyo in detail. Finally, it presents other gerontology related activities including the Gerontology Literacy Test started by the IOG in 2013.

